# Isolation of Protein-Associated Circular DNA from Healthy Cattle Serum

**DOI:** 10.1128/genomeA.00846-14

**Published:** 2014-08-28

**Authors:** Mathis Funk, Karin Gunst, Vincent Lucansky, Hermann Müller, Harald zur Hausen, Ethel-Michele de Villiers

**Affiliations:** a Division for the Characterization of Tumorviruses, Deutsches Krebsforschungszentrum, Heidelberg, Germany; b Institute for Virology, Faculty of Veterinary Medicine, University of Leipzig, Leipzig, Germany

## Abstract

Three replication-competent single-stranded DNA molecules sharing nucleotide similarity to transmissible spongiform encephalopathy (TSE)-associated isolate Sphinx 2.36 were isolated from healthy bovine serum.

## GENOME ANNOUNCEMENT

Nonpathogenic persistent virus infections of animals may prove pathogenic when transmitted to humans (1). We analyzed 120 serum samples collected from healthy cattle in an attempt to identify previously unknown viruses. Two isolation procedures were followed: initially, pools of 5 serum samples were subjected to OptiPrep (iodixanol) density gradient ultracentrifugation after benzonase treatment to remove all free DNA and RNA (2). Protein-associated DNA was extracted from fractions (Qiagen PCR purification kit) and 1 µl DNA/fraction subjected to rolling circle amplification (RCA) in a solution of 50 µM exonuclease-resistant random primers (Thermo Scientific), 3.2 µmol each deoxynucleoside triphosphate (dNTP) (TaKaRa), and 10 U phi29 polymerase (New England BioLabs). Restriction-digested products (EcoR1 or BamH1) were separated by agarose gel electrophoresis and cloned into vector pUC19. The sequencing of clones by primer walking revealed 2 sequences, those of HCBI1.225 (HCBI, healthy cattle blood isolate) (2,251 bp) and HCBI2.170 (1,407 bp), related to the transmissible spongiform encephalopathy (TSE)-associated circular DNA isolate Sphinx 2.36 (2,364 bp; accession no. HQ444405) (3). Subsequently, inverted PCR using primers designed on these 2 isolates was performed on DNA extracted from single serum samples, and the products were cloned into the vector pCR2.1 (Invitrogen). We failed to obtain the full-length genome sequences of HCBI1.225 and HCBI2.170. Inverted PCR using primers specific for Sphinx 2.36 (forward [nucleotides {nt} 2313 to 2336], 5′-CTAATGCAGATCAACACAGGGATA-3′, and reverse [nt 2312 to 2291], 5′-GAATTACAGGCTTTGCAATCTG-3′) resulted in one clone, HCBI7.228 (2,280 bp), sharing 78% similarity with Sphinx 2.36.

HCBI1.225 (2,251 bp) shares 81% nucleotide similarity with Sphinx 2.36 by BLASTn analysis, and HCBI2.170 (1,407 bp) shares 75% nucleotide similarity. HCBI1.225 contains 3 major open reading frames (ORFs). The putative protein (229 amino acids) predicted from the largest ORF was identified as a replication protein using ProtSweep (4) and shares 82% similarity by BLASTp analysis to the replicase 1-like protein (232 amino acids [aa]) of Sphinx 2.36. The other 2 putative proteins (127 aa and 111 aa) share 91% and 97% identity to the 96-aa and 124-aa proteins of Sphinx 2.36, respectively. The putative Rep proteins of the 3 isolates share between 81 and 87% similarity to Sphinx 2.36 and 86 and 93% between themselves in the overlapping regions.

The 3 isolates described here are, similarly to Sphinx 2.36, related to extrachromosomal DNA plasmids of *Acinetobacter*. The circular Sphinx 2.36 DNA was copurified with infectious particles in sucrose gradients (3). HCBI1.225 and HCBI2.170 were isolated from protein-associated gradient fractions after the removal of free DNA and RNA. The origin of these episomal DNA molecules remains to be elucidated.

### Nucleotide sequence accession numbers.

The complete sequences of HCBI1.225, HCBI2.170, and HCBI7.228 have been deposited in the EMBL Databank under accession numbers LK931499, LK931500, and LK931498, respectively.
